# Malondialdehyde and Neutrophil Gelatinase-Associated Lipocalin as Markers of Oxidative Stress in Small for Gestational Age Newborns from Hypertensive and Preeclamptic Pregnancies

**DOI:** 10.1155/2022/9246233

**Published:** 2022-02-18

**Authors:** Piotr Surmiak, Olga Wojnarowicz, Martyna Szymkowiak

**Affiliations:** ^1^Department of Neonatology, Faculty of Medical Sciences in Katowice, Medical University of Silesia in Katowice, Poland; ^2^Department of Neonatology, University Clinical Center Prof. K. Gibiński of Medical University of Silesia in Katowice, Poland

## Abstract

**Introduction:**

It is speculated that preeclampsia and hypertension during pregnancy are associated with an imbalance of the placental antioxidant defence, which results in the overproduction of reactive oxygen species and fetal growth restriction. Many research implied that oxidant stress in utero may be an important determinant of mortality and morbidity in neonates. Moreover, the authors demonstrated the reduced number of nephrons and a higher prevalence of renal injury in neonates with growth restriction, including small for gestational age (SGA) neonates. Alas, it remains unclear whether basal antioxidant status is altered in the kidneys of SGA newborns.

**Materials and Methods:**

In this study, we assessed neutrophil gelatinase-associated lipocalin (NGAL) and malondialdehyde (MDA) levels in samples collected from umbilical blood and 12 hours after delivery in neonates born by mothers suffering from preeclampsia or hypertension during pregnancy and those from physiological pregnancies. Additionally, the authors evaluated levels of the aforementioned biomarkers regarding the occurrence of growth restriction in newborns. For this study, we enrolled 27 newborns, which fulfilled inclusion criteria for SGA diagnosis (SGA group), while 21 were appropriate for gestational age neonates, as the AGA group.

**Results:**

In the presented study, we have found significant differences in umbilical cord MDA and NGAL concentration between the SGA and AGA groups. Such dependencies were not found in blood samples from neonates collected in the first 12 hours of life for MDA and NGAL concentrations. Additionally, we have observed differences in umbilical MDA and NGAL levels between newborns of preeclamptic or hypertensive mothers compared to healthy ones. A significant correlation between the occurrence of hypertension during pregnancy and umbilical MDA and NGAL concentrations was also found.

**Conclusions:**

Small for gestational age newborns or those born by preeclamptic and hypertensive mothers had significantly higher MDA and NGAL levels as compared to healthy ones. Further investigation is needed to understand the pathophysiologic influence of hypertension in pregnancy and oxidative stress injury in newborns with growth restriction.

## 1. Introduction

Oxidative stress may be defined as inadequate redox homeostasis, resulting from an intensified generation of reactive oxygen species (ROS). Certain gestational complications, such as hypertension or preeclampsia, seem to be associated with increased exposition to oxidative stress during and after delivery [1–3]. It is speculated that increased ROS generation has been implicated in intrauterine perturbations, like placental insufficiency, and may result in intrauterine growth restriction, observed in small for gestational age newborns (SGA) [4].

According to the current literature, hypertension and preeclampsia are described as one of the most common diseases of the gestation period and may affect even 10% of pregnancies worldwide; therefore, there is a dire need of finding biomarkers that would help define them in the early clinical stage.

One of the most frequently used biomarkers of oxidative stress is malondialdehyde (MDA), the main product of the lipid peroxidation process of polyunsaturated fatty acids [2].

Results of current studies have shown elevated MDA concentrations in preeclamptic women; therefore, increased lipid peroxidation is considered to be a causative factor for preeclampsia [2, 3]. In many studies, MDA overexpression was observed post cellular injury, resulting in the progress of tissue damage, which leads to vascular endothelial cell dysfunction [1, 2, 5]. Moreover, Li et al. presented a significant relation between MDA levels and the prevalence of kidney injury, as well as chronic kidney disease [6].

Available studies reported that neutrophil gelatinase-associated lipocalin (NGAL, lipocalin-2) seems to be the marker specific to altered renal function. The latest studies indicated NGAL use as a diagnostic and prognostic biomarker for acute kidney injury in the adult and pediatric population [7–9]. Authors suggested that NGAL increase may be characteristic for mothers with preeclampsia and their SGA infants alike [7, 8, 10, 11].

The present study focused on assessing NGAL and MDA levels in samples collected from the umbilical cord and 12 hours after delivery in neonates from pregnancies complicated by preeclampsia or hypertension. Moreover, the authors aimed to evaluate NGAL and MDA levels regarding the occurrence of growth restriction in SGA newborns.

## 2. Materials and Methods

### 2.1. Study Design, Settings, and Participants

This prospective, single-centre, case-control study included a total of 48 full-term and late preterm neonates, born at the Department of Neonatology, the University Clinical Center Medical University of Silesia in Katowice, in the period between April 1st, 2019, and April 1st, 2020. The study was approved by the appropriate Bioethics Committee of the Medical University of Silesia in Katowice (no. KNW/022/KB1/29/19). All the parents provided a signed informed consent before recruitment into the research.

The study group consisted of neonates that were small for gestational age, delivered by mothers with preeclampsia or hypertension during pregnancy. Into the research were qualified exclusively children born by caesarean section to avoid the nonspecific increase in the concentrations of the analyzed markers.

Among mothers eligible for the study, hypertension in pregnancy was diagnosed according to the guidelines of the Polish Society of Gynaecologists and Obstetricians as the increased blood pressure values above 135 mmHg for systolic pressure and/or higher than 85 mmHg for diastolic blood pressure, without accompanying proteinuria or other biochemical and haematological disorders [12]. Additionally, preeclampsia was recognised as hypertension, occurring after the 20th week of gestation, with proteinuria [1].

Newborns of diabetic mothers or multiple pregnancies, as well as extremely/very preterm (less than 32-week gestation) neonates and those with severe asphyxia and genetic and metabolic defects or with major blood group incompatibility, were excluded from the study.

Small for gestational age newborns were recognised based on clinical features of hypotrophy when birth weight was below the 10th percentile for gestational age as confirmed by the Fenton fetal-infant growth chart [13, 14].

In the whole investigated population, 27 newborns fulfilled inclusion criteria for SGA diagnosis (SGA group), while 21 were appropriate for gestational age; eutrophic neonates (AGA group) were enrolled in the study. Among SGA children, 12 were delivered by hypertensive and 6 by preeclamptic mothers. The remaining 9 cases were “constitutional SGA” infants, from otherwise healthy mothers.

Among patients in the AGA group, 6 were born from pregnancies complicated by hypertension and 15 newborns from physiological pregnancies. Therefore, all participants were divided into groups of newborns delivered by preeclamptic (*n* = 6), hypertensive (*n* = 18), and healthy mothers (*n* = 24) as a control group.

All children that were qualified for either study or control group had an ultrasound examination performed to exclude congenital malformations of the kidneys and urinary tract. Moreover, the onset of urination for all investigated neonates occurred in the first 12 hours of life and estimated diuresis values were age-appropriate.

The data on the mother-neonate dyads' demographics, clinical history, and relevant comorbidities were collected upon enrollment and are presented in Table 1.

### 2.2. Sample Collection and Storage

About 2 mL of arterial umbilical cord blood was withdrawn immediately after the placenta was delivered and after umbilical cord clamping from all participants. Directly after blood collection, blood-gas analysis was performed, and the remaining blood was centrifuged. Additionally, blood samples from newborns were collected from the peripheral vessels during a routine blood sampling procedure (including total blood count) in the first 12 hours of life. Obtained serum was frozen and stored at -80°C for later malondialdehyde and NGAL measurements.

### 2.3. Laboratory Measurements

Serum MDA concentration was determined by using enzyme-linked immunosorbent assay kit Cloud-Clone Corp. (Katy, TX, USA). The minimum detectable dose of malondialdehyde was typically less than 8.84 ng/mL.

For NGAL level measurements, Human Lipocalin-2/NGAL ELISA kit Biovendor (Brno, Czech Republic) was used. The limit of detection was calculated from the real lipocalin-2 values in wells and was 0.02 ng/mL. Blood-gas analysis was performed using the Siemens RAPIDPoint 500 Blood Gas System.

## 3. Statistical Analysis

Descriptive statistical analysis was performed to identify the incidence of growth retardation in newborns in our study group; baseline clinical characteristics were evaluated. Data were checked for normality of distribution using Shapiro-Wilk's test.

Due to nonparametrically distributed data, the quantitative variables were presented as medians with 95% confidence intervals (95% CI), whereas the qualitative variables were given as percentage values.

The statistical analysis was performed using the Kruskal-Wallis for multiple variables testing and the Mann–Whitney *U* test as well as Fisher's exact test between individual groups.

Additionally, MDA and NGAL plots were made of investigated groups of newborns to demonstrate the biomarker dynamics. To identify relations between analyzed biochemical markers, linear regression models were performed and presented as scatter plots.

Spearman's rank correlation was calculated for the laboratory measurements and clinical variables. All statistical analyses were done using STATISTICA version 13.3 (StatSoft Polska Inc.). In statistical analysis, *p* values less than 0.05 were considered statistically significant.

## 4. Results

**Figure 1:**
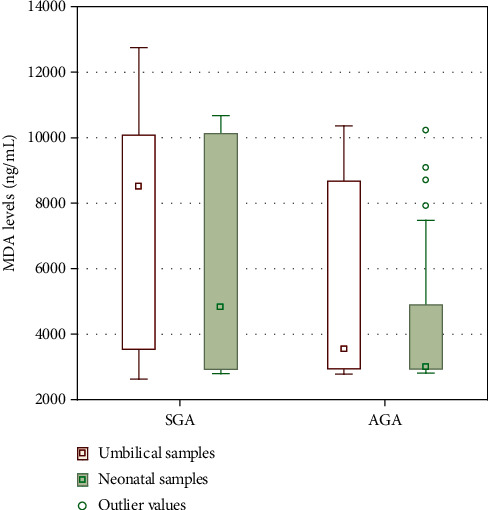
Malondialdehyde (MDA) levels in the umbilical cord and venous blood samples in the first 12 hours of small for gestational age (SGA) and adequate for gestational age (AGA) infants. Results are presented as medians and 95% confidence intervals with outlier values (^∗^).

**Figure 2:**
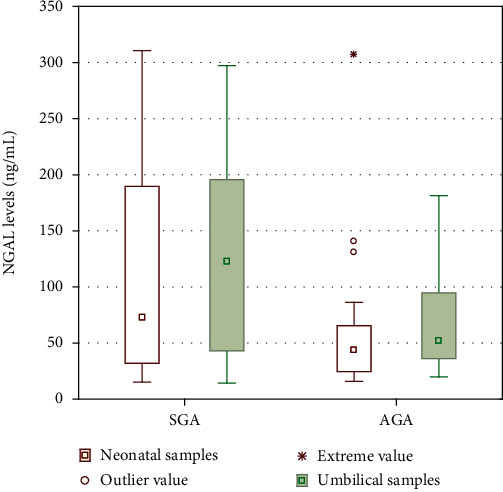
Neutrophil gelatinase-associated lipocalin (NGAL) levels in the umbilical cord and venous blood samples in the first 12 hours of small for gestational age (SGA) and adequate for gestational age (AGA) infants. Results are presented as medians and 95% confidence intervals with extreme and outlier values.

In the presented study, significant differences were found in umbilical cord MDA concentration between the SGA and AGA groups (8501.5 [3540.2-10079.5] vs. 3551.4 [2945.0-8675.8] ng/mL; *p* = 0.04). Similar findings were noticed for NGAL levels in umbilical cord samples (122.8 [43.0-195.6] vs. 52.1 [36.1-94.9] ng/mL; *p* = 0.02, respectively). Such dependencies were not found in blood samples from neonates collected in the first 12 hours of life for MDA levels (*p* = 0.15) as well as NGAL concentrations (*p* = 0.07). Results are presented in Figures 1 and 2.

While analyzing concentrations of individual markers in all participants of the study, we have found significant differences in umbilical levels of MDA and NGAL between neonates of preeclamptic or hypertensive mothers and those delivered by healthy mothers (*p* = 0.006 and *p* = 0.02, respectively). In blood samples, collected from newborns in the first 12 hours after birth from hypertensive and preeclamptic mothers, only NGAL concentrations were significantly increased in comparison to controls (*p* = 0.0001), as presented in Table 2.

Furthermore, MDA levels in blood samples collected 12 hours after delivery were elevated in preeclampsia and hypertension groups regarding neonates of healthy mothers; however, those findings were not statistically significant (*p* = 0.06).

Moreover, a significant correlation between maternal hypertension occurrence and MDA level in the umbilical cord blood (*r* = 0.44; *p* = 0.03) was determined. Additionally, we have found a correlation between the prevalence of hypertension during pregnancy and the concentration of umbilical NGAL (*r* = 0.38; *p* = 0.09).

We have not observed any significant relationships between umbilical cord lactate levels and MDA as well as NGAL concentrations (*p* > 0.05).

**Figure 3:**
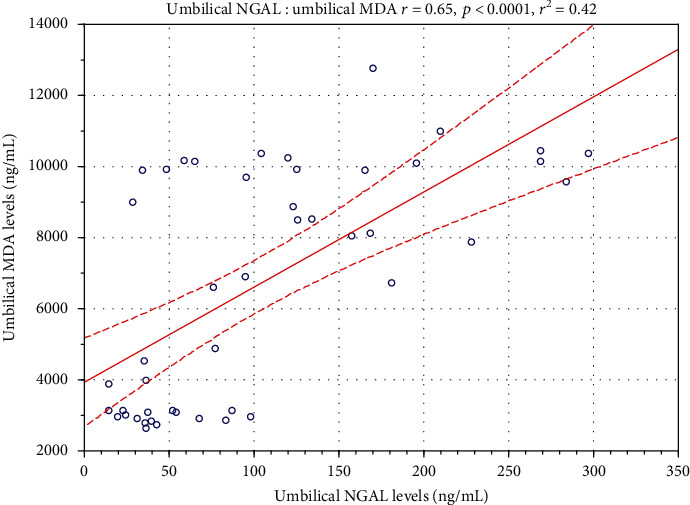
The statistical correlation between malondialdehyde (MDA) and neutrophil gelatinase-associated lipocalin (NGAL) concentrations in the umbilical cord of investigated neonates. Scatter plots depicting linear regression with confidence intervals for analyzed biochemical markers in the study population.

However, a strong positive correlation was found between MDA and NGAL levels in the umbilical cord (*r* = 0.65; *p* < 0.001), and the result of linear regressions for the aforementioned biomarkers are presented in Figure 3.

## 5. Discussion

In the presented study, we have evaluated the oxidative stress markers in the umbilical cord blood and the first 12 hours after delivery in small for gestational age newborns from pregnancies complicated by hypertension and preeclampsia. In the light of current knowledge on intrauterine growth restriction and its pathomechanism, placental insufficiency is considered a major factor in its aetiology [13, 15]. Placental dysfunction, as a consequence of an inadequate remodelling of uteroplacental circulation and ensuing hypoxia, may lead to vasoconstriction, reduced transfer of oxygen to the foetus and subsequent restriction of fetal growth [3, 15].

It is widely speculated in the literature that preeclampsia, as well as hypertension during pregnancy, is associated with an imbalance of the placental antioxidant defence, composed of the enzymatic and nonenzymatic defence systems and, therefore, with increased ROS synthesis in the placenta [3, 16, 17]. Available research provides studies that the concentration of malondialdehyde measured in umbilical cord blood was a reliant marker of perinatal oxidative stress [18, 19]. Shang et al. demonstrated significantly higher MDA levels in umbilical cord serum and placental tissue in pregnancies affected by oxidative stress [20]. Moreover, Weber et al. showed that elevated maternal oxidative stress correlated with increased occurrence of fetal growth restriction [21]. Those findings seem to concur with our study, in which we found significant differences in umbilical cord MDA levels between small for gestational age newborns and AGA ones. Dede et al. analyzed the levels of oxidative stress markers in the umbilical cord blood in SGA newborns reaching similar conclusions [22]. However, we have also measured MDA concentrations in blood samples collected in the first 12 hours of newborns' life, but we have not found such differences between those groups. Other authors connected increased levels of oxidative stress markers in the umbilical cord with the inadequate antioxidant defence of newborns with features of growth restriction [22]. In accordance with the available literature, we assume that decreasing levels of MDA in SGA newborns in the first 12 hours of life in comparison to high levels in the umbilical cord may be connected to either newborn using it in metabolic processes or malondialdehyde being rapidly metabolized by antioxidants, due to possible toxicity [4]. In light of the information above, arises a question of the placenta's protective role against MDA secretion or excretion in the fetoplacental circulation of SGA newborns, which would validate consideration of further research.

Many authors indicated a significant rate of short- and long-term complications among small for gestational age children, such as diabetes, coronary heart disease, or hypertension [13, 23].

In the pediatric population, elevated levels of malondialdehyde seem to be related to the occurrence of hypoxic-ischemic encephalopathy, indicating that free radical injury plays a major role in its causation [24].

Furthermore, intrauterine growth restriction seems to be related to underdeveloped kidneys with fewer functional nephrons, immature tubules, and abnormal glomeruli morphology which reduces kidney sufficiency. Such impairments of renal function, secondary to injury caused by hypoperfusion and oxidative stress, may negatively affect its hemodynamics, resulting in acute kidney injury, which correlates with higher morbidity and mortality rates later in life [25–28].

In our study, newborns from pregnancies complicated by preeclampsia and hypertension, including SGA, had significantly higher levels of NGAL both in the umbilical cord and in the first 12 hours of life. Moreover, MDA levels in the umbilical cord were also elevated in this population. Those results and significant correlation demonstrated between the concentrations of MDA and NGAL in the umbilical cord in the investigated group may indicate the influence of oxidative stress in the placenta on fetal organ development. Other researchers reached similar conclusions. Kamianowska et al. noticed increased urinary NGAL concentration in newborns with intrauterine growth restriction [25]. Furthermore, Soni et al. observed significantly smaller kidneys in the examined newborn piglets, additionally demonstrating elevated levels of MDA in renal tissue and NGAL in serum samples [26].

The kidneys seem to be extremely susceptible to ischemic-reperfusion injury, which occurs ensuing maternal hypertension and preeclampsia. Therefore, elevated NGAL levels were observed in pregnant women diagnosed with hypertension or preeclampsia and their children [7, 8]. Capelli et al. in their research presented higher urinary NGAL levels in infants with hypertensive mothers, indicating that hypertensive disorders of pregnancy may trigger a proinflammatory response in the offspring and negatively affect fetal organs [10].

In support of this hypothesis, in neonates born by hypertensive mothers elevated proinflammatory molecules, as a biomarker of gestational systemic inflammation, could reflect the impact of preeclampsia on the developing offspring and further health complications [10, 29–31].

## 6. Limitations

Due to the obstacles resulting from the ongoing COVID-19 pandemic restrictions, the number of participants in both the study and control groups was limited.

Perinatal asphyxia may be one of the major causative factors of oxidative stress. Previous studies confirmed the influence of acute perinatal asphyxia on an increase of both umbilical cord NGAL and MDA levels [9, 32].

Several studies have shown that excess body weight in pregnant women may be a cause of elevated levels of oxidative stress markers, both in mothers and in their offspring [29]. In our study, mothers' body weight and BMI were not taken into consideration, which may have affected the final results.

Other factors that may cause an increase in oxidative stress markers are maternal hyperglycaemia and maternal diabetes [20]. In accordance with those findings, maternal diabetes was one of the criteria excluding patients from our study.

## 7. Conclusions

Our research provided data that small for gestational age newborns or born by preeclamptic and hypertensive mothers had significantly elevated MDA and NGAL levels as compared to healthy ones. This may be confirmed by the influence of oxidative stress on the onset of subclinical altered organ function in growth-restricted neonates. Further investigation is needed to understand the pathophysiologic influence of hypertension in pregnancy on developing kidneys of foetuses with growth restriction.

## Figures and Tables

**Table 1:** Demographic data and perinatal characteristics of small for gestational age (SGA) and appropriate for gestational age (AGA) infants, as well as their mothers. Additionally, laboratory findings of blood gas analysis of the samples from the umbilical cord are also shown. Results are presented as medians and 95% confidence intervals or percentage values.

Investigated group	SGA group (*n* = 27)	AGA group (*n* = 21)	*p* value
Mothers			
(i) Age (years)	32 [28-33]	30 [27-31]	0.25
(ii) Primigravida (no.; %)	17; 63.0%	14; 66.7%	0.81
*Perinatal history*			
(i) Hypertension (no.; %)	12; 66.7%	6; 33.3%	0.04
(ii) Preeclampsia (no.; %)	6; 22.2%	0	0.44
(iii) Thyroid diseases (no.; %)	9; 33.3%	8; 38.1%	0.73
Neonates			
(i) Gestational age (weeks)	36 [36-37]	36 [35-38]	0.14
(ii) Gender: female (no.; %)	14; 51.9%	12; 57.1%	0.75
(iii) Birth weight (g)	2210.0 [1940.0-2340.0]	2450.0 [2150.0-2900.0]	0.05
*Postnatal complication*			
(i) Perinatal asphyxia (no.; %)	7; 25.9%	5; 23.8%	0.91
(ii) Hypothermia (no.; %)	18; 66.7%	6; 28.6%	0.04
(iii) Hypoglycemia (no.; %)	12; 44.4%	4; 19.0%	0.16
(iv) Respiratory disorder (no.; %)	7; 25.9%	3; 14.3%	0.45
(v) Peri-intraventricular			
Hemorrhage (no., %)	7; 25.9%	3; 14.3%	0.45
*Laboratory findings*			
(i) Lactate levels (mmol/L)	4.1 [3.1-4.6]	3.4 [2.6-5.1]	0.31
(ii) pH value < 7.2 (no.; %)	6; 22.2%	4; 19.0%	0.71

**Table 2:** Malondialdehyde (MDA) and neutrophil gelatinase-associated lipocalin (NGAL) levels in the umbilical cord and venous blood samples of newborns from preeclamptic, hypertensive groups as well as healthy ones as controls. Results are presented as medians and 95% confidence intervals.

Investigated group	Preeclampsia group (*n* = 6)	Hypertension group (*n* = 18)	Control group (*n* = 24)	*p* value
Umbilical MDA levels (ng/mL)	9096.0 [4875.4-10440.3]	8862.4 [3125.3-10128.4]	3129.1 [2942.8-6713.8]	0.006
MDA levels in the first 12 hours of life (ng/mL)	5848.5 [4210.6-7776.7]	5835.0 [2992.1-10189.6]	2974.2 [2902.4-3099.9]	0.06
*p* value	0.22	0.68	0.81	
Umbilical NGAL levels (ng/mL)	169.5 [77.2-195.6]	104.3 [52.1-157.8]	37.2 [34.6-83.6]	0.02
NGAL levels in the first 12 hours of life (ng/mL)	163.1 [64.3-235.5]	82.8 [49.1-154.1]	30.7 [22.3-42.9]	0.0001
*p* value	0.68	0.65	0.10	

## Data Availability

The data used to support the findings of this study are available from the corresponding author upon request.
